# Efficacy of Intra-Uterine Tamponade Balloon in Post-Partum Hemorrhage after Cesarean Delivery: An Impact Study

**DOI:** 10.3390/jcm10010081

**Published:** 2020-12-28

**Authors:** Mickaël Soued, Alexandre J. Vivanti, Daniel Smiljkovski, Xavier Deffieux, Alexandra Benachi, Agnès Le Gouez, Frédéric J. Mercier

**Affiliations:** 1 Department of Anesthesia, Hôpital Antoine Béclère, APHP, Université Paris Saclay, 157 rue de la Porte de Trivaux, 92140 Clamart, France; daniel.smiljkovski@gmail.com (D.S.); agnes.le-gouez@aphp.fr (A.L.G.); frederic.mercier@aphp.fr (F.J.M.); 2 Department of Gynecology and Obstetrics, Hôpital Antoine Béclère, APHP, Université Paris Saclay, 157 rue de la Porte de Trivaux, 92140 Clamart, France; alexandre.vivanti@aphp.fr (A.J.V.); xavier.deffieux@aphp.fr (X.D.); alexandra.benachi@aphp.fr (A.B.)

**Keywords:** post-partum hemorrhage, cesarean delivery, uterine balloon tamponade, embolization, hysterectomy

## Abstract

Invasive therapies (surgery or radiological embolization) are used to control severe post-partum hemorrhage. The intra-uterine tamponade balloon is a potential alternative, well documented after vaginal delivery. However, available data on its use after cesarean delivery remain scarce. This study assessed the efficacy of the intra-uterine tamponade balloon during post-partum hemorrhage in a cesarean delivery setting. Using a retrospective impact design, post-partum hemorrhage-related outcomes before (“pre-balloon” period) versus after implementation of intra-uterine tamponade balloon (“post-balloon” period) were compared. All women with post-partum hemorrhage requiring potent uterotonic treatment with prostaglandins after cesarean delivery over a 9-year period were eligible. The primary outcome was the rate of invasive procedure (conservative surgery, radiological embolization and/or hysterectomy). *p* < 0.05 was considered statistically significant. A total of 279 patients were included (140 vs. 139). Most baseline characteristics were comparable between the two studied periods. The success rate of the intra-uterine tamponade balloon was 82%, and no related complications occurred. Rates of invasive procedures and transfusion were significantly reduced (28.6% vs. 11.5%, *p* < 0.001 and 44.3% vs. 28.1%, *p* = 0.006 respectively) during the “post-balloon” period, and length of hospital stay was shorter (*p* < 0.001). Implementation of intra-uterine tamponade balloon during post-partum hemorrhage after cesarean delivery appears to be safe and effective, with a decrease in both invasive procedures and transfusion rates.

## 1. Introduction

Post-partum hemorrhage (PPH) remains a major cause of maternal death [1], with uterine atony implicated in up to 80% of all bleedings [2,3]. First-line treatment includes oxytocin followed, when needed, by prostaglandins. In case of failure of these first-line treatments, second-line invasive therapies such as artery ligation [4,5], uterine compressive suture [6,7], and/or radiological artery embolization have to be implemented [8]. Each of these treatments can be performed alone or they may be also combined, with failure leading to hysterectomy. Because of the invasive character and inherent morbidity of these second-line therapies [9,10,11], alternative approaches have been suggested: the Blakemore probe [12,13] first, and then a few other devices, among which the intra-uterine tamponade balloon (IUTB) has triggered much interest [14,15,16].

In the last 20 years, several studies have documented and highlighted the usefulness and safety of IUTB in PPH after vaginal delivery by showing decreased requirements for invasive procedures, promoting less morbidity and/or less subsequent fertility impairment. This has led to positioning this device prior to invasive procedures (surgery or embolization) in PPH algorithms and guidelines after vaginal delivery in many countries [17,18,19]. However, there is a much lower level of evidence for the efficacy of IUTB when used in a cesarean delivery setting. This may probably be explained by the fear of damaging the brand-new uterine scar while inserting the IUTB. This could also be related to the fact that operators may prefer an immediate invasive surgical procedure during or immediately after cesarean delivery. Consequently, the place and the timing of IUTB insertion after cesarean delivery remain unclear. Thus, our impact study aimed to evaluate the benefits and the complications rate of IUTB use in this cesarean delivery setting.

## 2. Materials and Methods

We conducted a monocentric retrospective impact study in the maternity unit of the tertiary academic Antoine Béclère Hospital (3600 deliveries per year) using a controlled “before” vs. “after” study design. To evaluate the value of IUTB in PPH after cesarean delivery, we compared all different PPH-related outcomes before vs. after implementation of IUTB. The first period, before implementation of IUTB, was defined as the controlled “pre-balloon” period, and the next as the “post-balloon” period. All women with PPH after cesarean delivery requiring a switch from oxytocin to prostaglandins were eligible, and exhaustively compiled in the database. In France, this switch is recommended within no more than 30 min for persistent PPH, and the prostaglandin used is sulprostone (Nalador^®^, BAYER Healthcare, Loos, France) [17].

**Figure 1:**
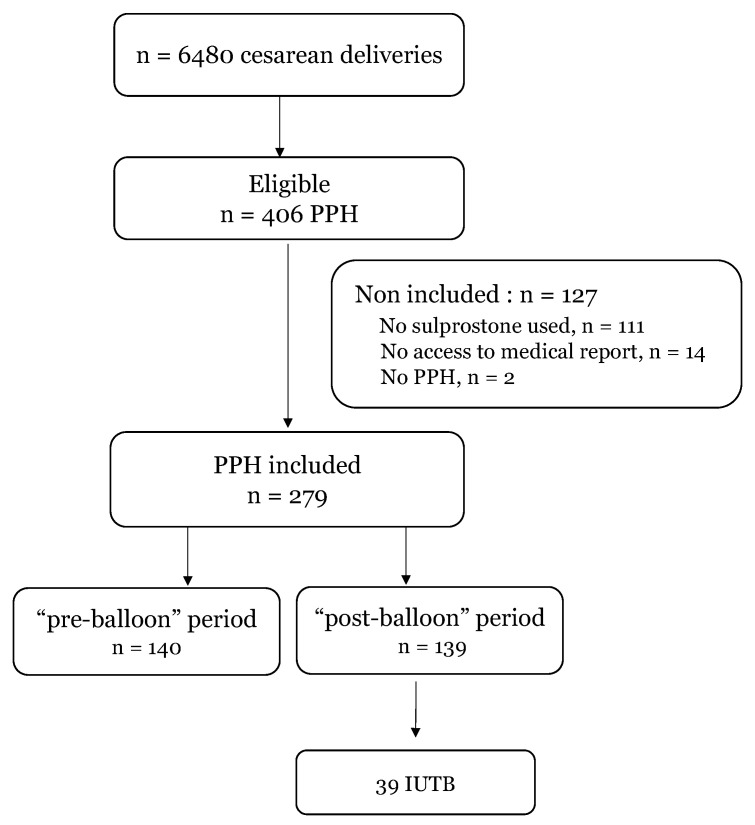
Flow chart representing eligible, included and non-included patients. PPH: post-partum hemorrhage; IUTB: intra-uterine tamponade balloon.

Data were extracted from a local computerized database (DIAMM^®^ software v8.6r0 -Micro 6^®^, Villers Les Nancy, France) by gradually searching with the following keywords: “Post-Partum Hemorrhage” AND “cesarean” AND/OR “nalador” AND/OR “sulprostone” AND/OR “intra-uterine tamponade balloon” AND/OR “bakri” (Figure 1). In our institution, after extracting data from DIAMM^®^ software, it appeared that the IUTB started to be used for PPH following cesarean delivery on 18 December 2013. Therefore, the “after” period (“post-balloon” period) was defined from 1 December 2013 onwards, and until 31 March 2018. The controlled “before” period (“pre-balloon” period) was defined symmetrically as the 52 months that elapsed between 1 August 2009 and 30 November 2013.

During both periods, no major modifications occurred in our PPH protocol. Our protocol consists in prophylactic administration of either oxytocin or carbetocin just after delivery. In case of primary PPH (defined as blood loss > 500 mL during the first 24 h following cesarean delivery [17]), sulprostone was started intravenously at a dose rate of 500 μg/h for 1 h, followed when needed by a continuous infusion at 100 μg/h [17]. Tranexamic acid was also administered concomitantly. When PPH was still active despite this treatment, an invasive procedure was considered (usually between 30 to 60 min after onset of sulprostone infusion) and was performed by the attending obstetrician, using artery ligation, embolization, compressive uterine suture and/or hysterectomy when needed. In the post-balloon period, during which the IUTB had become available, the attending obstetrician could also consider using it. In our institution, the Bakri balloon was the only IUTB available. IUTB insertion and inflation were carried out according to the manufacturer’s procedure [20]. The timing of IUTB insertion was left to the obstetrician’s discretion.

The characteristics of the women included in the study were collected: date of delivery, age, height and weight at delivery, gestational age, gestity before delivery, multiple pregnancy, preeclampsia, history of scarred uterus, history of PPH, elective setting for the cesarean delivery, preoperative hemoglobin concentration, birth weight (Z-scores) in singleton pregnancies and placentation abnormality. Insertion (previa) and invasion (accreta, increta and percreta) placentation abnormalities were recorded.

The following data related to PPH were also collected: the total dose of sulprostone infused, transfusion requirement with details of each blood product (red blood cells (RBC), fresh frozen plasma (FFP), concentrates of platelets units (CPU)), tranexamic acid, fibrinogen concentrates, recombinant activated factor VII (rFVIIa) use and invasive procedures with details (ligation, compressive suture, embolization or hysterectomy). Artery ligation and compressive uterine suture were defined as conservative surgical procedures (CSP). We defined as “intraoperative” each procedure performed during the cesarean delivery. Procedures were considered “postoperative” if performed after the first surgery (i.e., the cesarean delivery). Type of anesthesia, sepsis occurring during hospitalization, high dependency unit (HDU) requirements (in intensive care or post-anesthesia care unit for 24 h at least) with length of stay, overall length of hospital stay and maternal mortality data were also collected.

Finally, we collected data related to IUTB: success rate (defined as no need for additional invasive hemostatic interventions), timing of insertion, inflated volume, duration of use, maximum pain scores, analgesia provided and potential complications. The timing of insertion was defined as “intraoperative” or “postoperative” as for invasive procedures.

Our primary outcome was the rate of second-line invasive procedures (CSP, radiological artery embolization and/or hysterectomy). Secondary outcomes were other main factors related to PPH severity: transfusion rate, sepsis, HDU requirements, length of hospital stay and maternal mortality.

Parametric distribution of each variable was assessed by the Shapiro–Wilk test and homogeneity of variances was checked. Because some studied variables were normally distributed whereas others were not, continuous data were expressed, respectively, as mean (standard deviation) or median (interquartile range) as appropriate. Categorical variables were expressed as a number (percentage). Comparisons between “pre-balloon” and “post-balloon” periods were performed by Chi 2 test or Fisher exact test, Mann–Whitney or Student t-test as appropriate. Odds ratios with their 95% confidence intervals (95 CI) were also calculated. A *p*-value < 0.05 was considered statistically significant. All statistical analyses and graphs were performed using R software (Version 3.5.1, R Core Team (2018), Vienna, Austria, https://www.R-project.org/) with “Epi”, “questionr” and “GGplot” packages.

As requested by national regulations, the database was anonymized with numeric codes to prevent risk of patients’ identification and leakage. The Ethical Committee of the French Society of Anesthesia and Intensive Care approved the study on June 3, 2018 (IRB number 00010254-2018-072) and waived the requirement for written informed consent (non-interventional retrospective impact study).

## 3. Results

Among 6480 cesarean deliveries screened, 406 cases of PPH were eligible and 279 cases of PPH were included. Among the 127 cases of PPH not included, there were 111 cases for which no sulprostone was used, 14 cases of PPH with a medical report not fully accessible and 2 cases with no PPH finally retrieved after detailed examination of their charts (Figure 1).

Baseline characteristics are displayed in Table 1. There were no differences between “pre-balloon” and “post-balloon” groups regarding most characteristics. However, there were statistically significant but mild clinical differences regarding body mass index and gestational age at delivery; in addition, abnormal placental invasion was more frequent in the “pre-balloon” group than in the “post-balloon” group (7.9% vs. 1.4%, *p* = 0.02). There was also a trend of lower preeclampsia frequency during the “post-balloon” period (Table 1).

### 3.1. Invasive Procedures

The overall rate of any invasive procedures was significantly higher in the “pre-balloon” period compared to the “post-balloon” period (28.6% vs. 11.5%, *p* < 0.001). This was related to a higher rate of conservative surgical procedure in the “pre-balloon” period (25.0% vs. 10.8%, *p* = 0.003), with no statistically significant difference regarding the rates of radiological embolization and hysterectomy (4.3% vs. 0.7% (*p* = 0.12) and 9.3% vs. 4.3% (*p* = 0.16), respectively) (Table 2).

**Figure 2:**
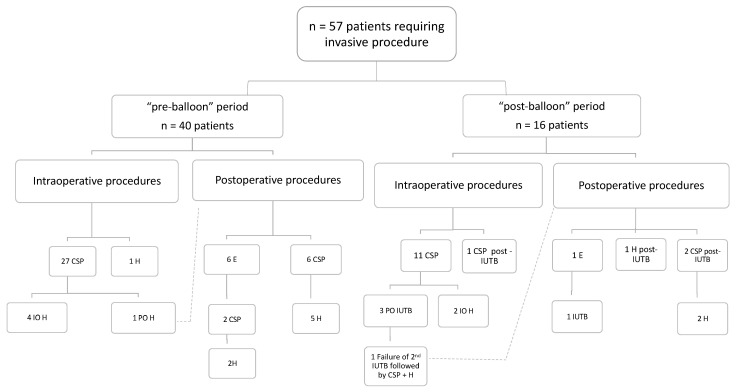
Flow chart representing the timing (intraoperative/postoperative) at which each invasive procedure (CSP, E and/or H) was performed. CSP: conservative surgical procedure; H: hysterectomy; E: uterine artery embolization; IO: intraoperative; PO: postoperative. In one patient for each period, an invasive procedure was used intraoperatively and another one followed postoperatively (a dash line links both procedures). Of note, the detailed use of the intra-uterine tamponade balloon (IUTB) during the “post-balloon” period and its timing versus the invasive procedure is displayed in Figure 3.

The timing at which invasive procedures were performed is illustrated in Figure 2. No complications related to invasive procedures were retrieved.

### 3.2. Transfusion

Overall transfusion rate was also higher prior to IUTB introduction (44.3% vs. 28.1%, *p* = 0.006). Patients had significantly lower transfusion rates in the “post-balloon” period for RBC (OR = 0.45 (0.27–0.75), *p* = 0.002), FFP (OR = 0.38 (0.22–0.67), *p* < 0.001), CPU (OR = 0.35 (0.15–0.81), *p* = 0.02) and rFVIIa (OR = 0.15 (0.03–0.67), *p* = 0.006), but not for fibrinogen concentrates (OR = 0.64 (0.33–1.25), *p* = 0.25). The amount of RBC, FFP, CPU and rFVIIa transfused were also lower (Table 3).

### 3.3. Secondary Outcomes

Post-partum sepsis incidence was similar between the two periods (7.5% vs. 8.5%, *p* = 0.82), but women were more likely to be transferred to HDU (15.6% vs. 7.2%, *p* = 0.05) during the “pre-balloon” period. The median (IQR) length of stay in HDU was similar between the two periods (2.0 days (1.0–3.0) vs. 2.5 days (1.25–3.75), *p* = 0.67). Nonetheless, the overall length of hospital stay was significantly higher in the “pre-balloon” period (7.0 days (6.0–9.0) vs. 6.0 days (5.0–7.0), *p* < 0.001). One maternal death was observed during the “pre-balloon” period (0.7% vs. 0%, *p* = 1.0).

### 3.4. IUTBs Characteristics

**Figure 3:**
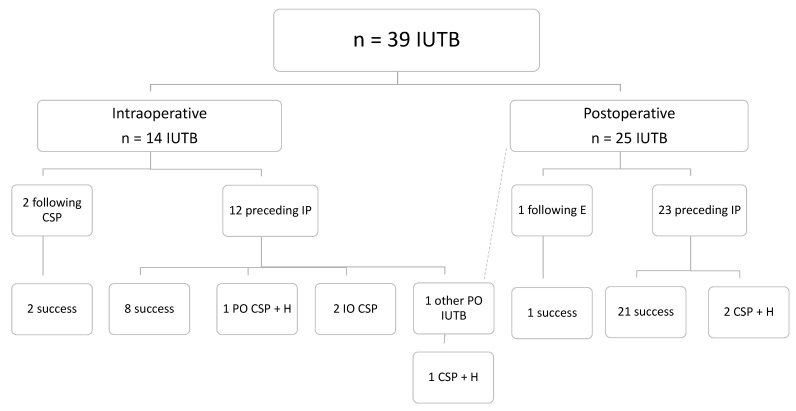
Flow chart representing the timing (intraoperative/postoperative) at which each intra-uterine tamponade balloon was used (thus, solely during the “post-balloon” period). IUTB: intra-uterine tamponade balloon; CSP: conservative surgical procedure; IP: invasive procedure; E: uterine artery embolization; H: hysterectomy; IO: intraoperative; PO: postoperative.

There were 39 cases of IUTB insertion, and one patient received 2 IUTBs. A total of 14 IUTBs were used intraoperatively (36%) and 25 postoperatively (64%). In 35 cases, IUTBs were used prior to invasive procedures (Figure 3).

The overall IUTB success rate was 82% (32 of 39 cases). Among 14 intraoperative insertions of IUTB, 10 (71%) were successful (2 of them made after conservative surgery procedures). Among 25 postoperative IUTB insertions, 22 (88%) were successful, while 3 were unsuccessful and were followed by hysterectomy. One successful insertion was performed after radiological embolization. All characteristics related to inflated volumes, duration of use, pain scores and analgesia provided are summarized in Table 4. No complications related to IUTB were retrieved.

### 3.5. Sensitivity Analysis without Patients with Morbidly Adherent Placenta

Among the 13 PPH cases (11 in the “pre-balloon” period and 2 in the “post-balloon” period, Table 1) that occurred in women with placental invasion abnormalities, 9 required an invasive procedure in the “pre-balloon” period and 2 in the “post-balloon” period. None of these patients experienced IUTB insertion. Morbidly adherent placenta is known to be a PPH risk factor, and our group comparability highlighted a higher rate of patients with placenta accreta spectrum in the “pre-balloon” period (Table 1). Thus, we performed a sensitivity analysis by removing these 13 patients and found no significant modifications in our results (Tables S1–S3, Supplementary files).

## 4. Discussion

This controlled before-and-after impact study showed a significant reduction in second-line invasive procedure rate (OR 0.3 (0.17-0.61)) after IUTB implementation for PPH management during cesarean delivery. This reduction involved mainly CSPs (25.0% vs. 10.8%, *p* = 0.003), which are the invasive surgical procedures most often used for intraoperative bleeding, but a similar trend was also observed for embolization and hysterectomies (Table 2).

Currently, only a few studies have evaluated the efficacy of IUTB following cesarean delivery, and they have focused on placenta praevia [21,22,23,24]. Thus, the literature on unselected population after cesarean delivery remains poor. In the two previous available impact studies that were positive on IUTB use [25,26], the vast majority of the PPH populations had delivered vaginally, whereas patients undergoing cesarean deliveries were solely a small subset with no conclusive effects. Two cohort studies have enrolled a significant number of patients with PPH receiving IUTB with a good success rate after both cesarean deliveries and vaginal deliveries [27,28]. However, these studies were purely descriptive series, i.e., without a historical control group prior to IUTB implementation. Another study conducted by Lo et al. [29] reported a significant reduction of compressive uterine suture and hysterectomy after IUTB implementation, but again, patients with cesarean delivery represented only a subgroup of the population studied. Barinov et al. [30] also showed that IUTB could significantly reduce the number of hysterectomies after PPH occurring in a cesarean delivery setting. However, IUTB was not implemented alone; it was part of a combined strategy, which also included an early surgical hemostasis and thromboelastographic assessment of coagulation. More recently, Revert et al. [31] have evaluated the impact of IUTB implementation in a large population by comparing two maternity unit networks, one using IUTB but not the other. After adjusting for confounding factors, they found in the network using IUTB a lower risk of invasive procedures among women who delivered vaginally, but not among women that had cesarean delivery [31]. Therefore, our study provides the strongest evidence to date for a significant reduction in invasive procedure requirements during PPH, following IUTB availability and use in the specific cesarean delivery setting. Differences between our results and those published by Revert et al. [31] could be explained by maternity unit heterogeneity in the latter study, with variability in local protocols; these differences might also be partly related to heterogeneity in IUTB inserted (mix of Bakri and Belfort–Dildy balloons).

We found a global rate of IUTB success of 82% (32/39), which is in agreement with most of the previous literature [25,26,27,29]. In the “post-balloon” period, we used more intraoperative IUTB than intraoperative invasive procedures (*n* = 14 vs. 12, Figure 2; Figure 3) and 10 IUTB were successful, suggesting that they could often be a suitable alternative to invasive procedures intraoperatively. Along the same line, we also found that 22 out of 25 postoperative IUTB insertions were successful (Figure 3), which similarly suggests the advantage of using IUTB postoperatively as a first option to avoid moving a potentially unstable patient back to the operating theater or to the radiology department for an invasive procedure.

More and more observational series also document that IUTB can be safely used in a cesarean delivery setting [21,22,24,32]. However, although we also did not find any complications with IUTB, such devices must be used carefully, because some case reports have described IUTB migrations through the uterus [33,34,35]. Thus, further studies are still needed to determine precise optimal timing and indication of IUTB insertion after cesarean delivery, as an alternative or as a supplement to invasive procedure.

An important strength of our impact study is related to the population characteristics between the two studied periods. Indeed, the incidence of PPH after cesarean delivery was similar in the controlled “pre-balloon” versus “post-balloon” periods (*p* = 0.43), and most of the women’s characteristics were also comparable regarding PPH risk factors [3]. Differences in body mass index (BMI) (27.6 vs. 28.8) and gestational age (38.3 vs. 39.0 weeks), although statistically significant, do not appear to be clinically relevant and, if so, would tend to increase PPH incidence in the “post-balloon” period and thus to reinforce the study results. Discrepancies in number of patients with placenta accreta between the two periods are not due to a change in patients’ recruitment, but rather to a change in management policy of placenta accreta. Indeed, in the meantime, we implemented new French [36] and international PPH guidelines [37,38] that promoted in this placenta accreta setting either non-conservative surgery (cesarean hysterectomy) or conservative management (placenta left in situ) rather than attempting forced placenta removal [39] as formerly. Furthermore, most of these patients in the “post-balloon” period did not meet our PPH inclusion criteria that involved the use of sulprostone, because this potent uterotonic is not recommended when the adherent placenta is left in situ or when cesarean hysterectomy is planned at once. Another strength of this study is to provide information on the timing of IUTB use, and thus to show that its efficacy appears high in both the intraoperative and postoperative periods.

Our study also has some limitations. First, in “before-and-after” impact studies, changes in routine practices can occur and produce biases, such as dropout threat, maturation threat, testing threat and history threat. However, apart from the implementation of IUTB, no modifications in our PPH protocol occurred except for the 13 patients with placenta accreta, and a sensitivity analysis excluding these patients did not change our results. Even if randomized controlled trials are desirable to formally establish a causal link, impact studies are also very useful in providing detailed information on a new intervention in a real-life setting.

Another limitation could be the absence of blood loss assessment or calculation. Formulae may be used [40,41], but only for patients without major modification of plasma volume (which pregnant women have). Postoperative hemoglobin concentration [25] (especially after RBC transfusion), estimated visual blood loss [28] or even blood loss measurements (with risk of amniotic fluid contamination) may also be poorly reliable, particularly in retrospective studies. The significant reduction in transfusion requirements we documented likely reflects better the severity and the economic impact of PPH.

The timing of IUTB insertion was left to the discretion of the obstetrician. This attitude could be responsible for a moderate inter-operator variation in IUTB use, although PPH management protocols in our maternity unit tend to promote homogeneous obstetric attitudes.

Lastly, even if this series (*n* = 279) remains the larger available today in cesarean delivery setting, the monocentric design may limit its external validity.

## 5. Conclusions

In conclusion, the implementation of IUTB in our PPH protocol for cesarean delivery was associated with a drastic drop in second-line invasive procedures. Our results support the effective and safe use of this device to prevent potential morbidity associated with radiological embolization, artery ligations and/or emergency hysterectomy.

## Figures and Tables

**Table 1:** Detailed characteristics of patients with PPH included.

Characteristics	“Pre-Balloon” (*n* = 140 PPH)	“Post-Balloon” (*n* = 139 PPH)	*p*
PPH requiring sulprostone during CD ^1^ among all CD over the studied period, *n* (%)	140 (4.5)	139 (4.1)	0.43
Age (years), mean ± SD	33.6 ± 5.9	34.0 ± 5.7	0.60
Body mass index (kg/m2), median (IQR)	27.6 (24.7–30.3)	28.8 (25.8–32.6)	0.01
Gestational age (weeks), median (IQR)	38.3 (35.6–40.0)	39.0 (36.9–40.6)	0.02
Gestity, median (IQR)	2.0 (1.0–3.0)	2.0 (1.0–3.0)	0.75
Multiple pregnancies, *n* (%)	36 (25.9)	28 (20.1)	0.32
History of uterine surgery, *n* (%)	42 (30.2)	46 (33.3)	0.67
History of PPH, *n* (%)	12 (8.6)	6 (4.3)	0.22
History of PPH requiring transfusion, *n* (%)	10 (7.1)	4 (2.9)	0.17
Placentation abnormality, *n* (%)			
Insertion	15 (10.7)	12 (8.6)	0.7
Invasion	11 (7.9)	2 (1.4)	0.02
Elective CD, *n* (%)	34 (24.6)	29 (20.9)	0.54
Preeclampsia, *n* (%)	25 (17.9)	13 (9.4)	0.06
Preoperative Hb concentration in g/dL, mean ± SD	11.8 ± 1.3	11.8 ± 1.1	0.71
Sulprostone infused, (vials of 500 μg), median (IQR)	2.0 (2.0–2.0)	2.0 (2.0–2.0)	0.31
Administration of tranexamic acid, *n* (%)	89 (66.9)	98 (74.8)	0.20
Macrosomia, *n* (%)	3 (2.1)	9 (6.5)	0.08
Z-score (singleton pregnancies), mean ± SD	−0.022 ± 1.1	0.06 ± 1.2	0.4
Type of anesthesia			
Epidural, *n* (%)	45 (32.4)	60 (43.2)	0.08
Combined spinal-epidural, *n* (%)	35 (25.2)	24 (17.3)	0.14
Spinal, *n* (%)	8 (5.8)	17 (12.2)	0.09
General, *n* (%)	51 (36.7)	38 (27.3)	0.13

^1^ Cesarean delivery.

**Table 2:** Rates of the various invasive procedures implemented. Related odds ratios are expressed with their 95% confidence intervals.

Outcome, n (%)	“Pre-Balloon” (*n* = 140)	“Post-Balloon” (*n* = 139)	Odds Ratio (95 CI)	*p*
Patients with any invasive procedure *	40 (28.6)	16 (11.5)	0.3 (0.17–0.61)	<0.001
Details of the invasive procedures				
Conservative surgical procedure	35 (25.0)	15 (10.8)	0.36 (0.19–0.70)	0.003
Embolization	6 (4.3)	1 (0.7)	0.16 (0.02–1.36)	0.12
Hysterectomy	13 (9.3)	6 (4.3)	0.44 (0.13–1.3)	0.16

* primary outcome. CI: Confidence Interval.

**Table 3:** Requirement rates of transfusion with details by blood product. Related odds ratios are expressed with their 95% confidence intervals.

Transfusion Outcomes	“Pre-Balloon” (*n* = 140)	“Post-Balloon” (*n* = 139)	Odds Ratio (95 CI)	*p*
Overall transfusion rate, *n* (%)	62 (44.3)	39 (28.1)	0.49 (0.3–0.8)	0.006
Red blood cells units, *n* (%)	61 (43.6)	36 (25.9)	0.45 (0.27–0.75)	0.002
Fresh frozen plasma units, *n* (%)	51 (36.4)	25 (18.0)	0.38 (0.22–0.67)	<0.001
Concentrates of platelets units, *n* (%)	21 (15.0)	8 (5.8)	0.35 (0.15–0.81)	0.02
Fibrinogen concentrates, *n* (%)	26 (18.6)	17 (12.8)	0.64 (0.33–1.25)	0.25
Recombinant activated factor VII (rFVIIa), *n* (%)	13 (9.3)	2 (1.5)	0.15 (0.03–0.67)	0.006

**Table 4:** Detailed characteristics of all patients who benefited from IUTB. Success was defined as no other hemostatic interventions needed.

Characteristics	*n* = 39 IUTB
Placental insertion abnormality, *n* (%)	5 (12.8)
Intraoperative insertion, *n* (%)	14 (36%)
Balloon inflated volume in mL, mean ± SD	332 ± 107
Duration of use in hours, mean ± SD	14.9 ± 6.4
Maximum numeric pain score (0–10), median (IQR)	4.0 (0–6)
Analgesia provided, *n* (%)	
Epidural	16 (41.0)
General anesthesia	7 (17.9)
Morphine patient-controlled analgesia	9 (23.1)
TAP block	2 (5.1)
Other analgesics	2 (5.1)
None	3 (7.7)
Overall success rate, *n* (%)	32 (82%)

## Data Availability

The data presented in this study are available on request from the corresponding author. The data are not publicly available due to privacy protection.

## Supplementary Materials

The following are available online at https://www.mdpi.com/2077-0383/10/1/81/s1, Table S1: Detailed characteristics of patients without placenta accreta spectrum (13 patients overall excluded: 11 in the “pre-balloon” period, 2 in the “post-balloon” period), Table S2: Rates of the various invasive procedures implemented (when 13 patients with placenta accreta spectrum were excluded: 11 in the “pre-balloon” period, 2 in the “post-balloon” period, Table S3: Requirement rates of transfusion with details by blood product (when 13 patients with placenta accreta spectrum were excluded: 11 in the “pre-balloon” period, 2 in the “post-balloon” period).
