# Supplementary material for: Efficacy of Intra-Uterine Tamponade Balloon in Post-Partum Hemorrhage after Cesarean Delivery: An Impact Study
Source: J Clin Med. 2020 Dec 28;10(1):81. doi: 10.3390/jcm10010081 (PMC7795364; doi:10.3390/jcm10010081)
Supplement: Supplementary file 1 [file jcm-10-00081-s001.pdf]

Supplementary Materials

Table S1. Detailed characteristics of patients without placenta accreta spectrum (13 patients overall excluded: 11 in the "pre-balloon" period, 2 in the "post-balloon" period).

Characteristics	"Pre-balloon" (n = 129 PPH)	"Post-balloon" (n = 137 PPH)	*p*
PPH requiring sulprostone during CD[1] among all CD over the studied period, n (%)	129 (4.2)	137 (4.0)	0.84
Age (years), mean ± SD	33.1 ± 5.8	33.9 ± 5.7	0.26
Body mass index (kg/m2), median (IQR)	28.0 (24.8–30.8)	28.8 (25.8–32.6)	0.04
Gestational age (weeks), median (IQR)	38.3 (35.6–40.0)	39.0 (36.0–40.6)	0.03
Gestity, median (IQR)	2.0 (1.0–3.0)	2.0 (1.0–3.0)	0.61
Multiple pregnancies, n (%)	34 (26.6)	28 (20.4)	0.30
History of uterine surgery, n (%)	38 (29.7)	44 (32.4)	0.74
History of PPH, n (%)	9 (7.0)	5 (3.6)	0.34
History of PPH requiring transfusion, n (%)	7 (5.4)	3 (2.2)	0.21
Placentation abnormality, n (%)			
Insertion	15 (11.6)	12 (8.8)	0.57
Elective CD, n (%)	31 (24.4)	28 (20.4)	0.53
Preeclampsia, n (%)	22 (17.1)	13 (9.5)	0.1
Preoperative Hb concentration in g/dL, mean ± SD	11.8 ± 1.3	11.9 ± 1.1	0.68
Sulprostone infused, (vials of 500 µg), median (IQR)	2.0 (2.0–2.0)	2.0 (2.0–2.0)	0.33
Administration of tranexamic acid, n (%)	82 (66.1)	97 (75.2)	0.15
Macrosomia, n (%)	3 (2.3)	9 (6.6)	0.14
Z-score (singleton pregnancies), mean ± SD	-0.02 ± 1.1	0.04 ± 1.2	0.69
Type of anesthesia			
Epidural, n (%)	44 (34.1)	60 (43.8)	0.14
Combined spinal-epidural, n (%)	34 (26.4)	24 (17.5)	0.1
Spinal, n (%)	8 (6.2)	17 (12.4)	0.13
General, n (%)	43 (33.3)	36 (26.3)	0.26

[1] Cesarean delivery. PPH: Post-partum hemorrhage.

Table S2. Rates of the various invasive procedures implemented (when 13 patients with placenta accreta spectrum were excluded: 11 in the "pre-balloon" period, 2 in the "post-balloon" period).

Outcome, n (%)	"Pre-balloon" (n = 129)	"Post-balloon" (n = 137)	Odds ratio (95 CI)	*p*
Patients with any invasive procedure	32 (24.8)	14 (10.2)	0.35 (0.17–0.68)	0.002
Details of the invasive procedures				
Conservative surgical procedure	24 (18.6)	12 (8.8)	0.42 (0.20–0.88)	0.03
Embolization	5 (3.9)	1 (0.7)	0.18 (0.02–1.58)	0.11
Hysterectomy	8 (6.2)	4 (2.9)	0.45 (0.13–1.55)	0.24

Related odds ratios are expressed with their 95% confidence intervals (CI).

Table S3. Requirement rates of transfusion with details by blood product (when 13 patients with placenta accreta spectrum were excluded: 11 in the "pre-balloon" period, 2 in the "post-balloon" period).

Transfusion outcomes	"Pre-balloon" (n = 129)	"Post-balloon" (n = 137)	Odds ratio (95 CI)	*p*
Overall transfusion requirement, n (%)	54 (41.9)	37 (27.0)	0.51 (0.31–0.86)	0.02
Red blood cells units, n (%)	53 (41.1)	34 (24.8)	0.47 (0.28–0.80)	0.007
Fresh frozen plasma units, n (%)	44 (34.1)	23 (16.8)	0.39 [0.22–0.69]	0.002

Concentrates of platelets units, n (%)	18 (14.0)	8 (5.8)	0.38 (0.16–0.91)	0.04
Fibrinogen concentrates, n (%)	19 (14.7)	15 (11.5)	0.75 (0.36–1.55)	0.54
rFVIIa, n (%)	10 (7.8)	2 (1.5)	0.18 (0.04–0.86)	0.02

Related odds ratios are expressed with their 95% confidence intervals (CI).
